# Comparison of measured and predicted mesiodistal tooth-widths of 13–17 years old Kenyans: a descriptive cross-sectional study to develop a new prediction equation for use in the mixed dentition in a Kenyan population

**DOI:** 10.1186/s12903-022-02368-y

**Published:** 2022-08-09

**Authors:** Nduguyu Kerre, James Lwanga Ngesa, Peter Ng’ang’a, Arthur Musakulu Kemoli, Janella Bermudez, Ana Lucia Seminario

**Affiliations:** 1 Orthomax Dental Centre, Nairobi, Kenya; 2 Department of Dental Sciences, University of Nairobi, Nairobi, Kenya; 3 City Dental Clinic, Nairobi, Kenya; 4 Department of Dental Sciences, University of Nairobi, Nairobi, Kenya; 5 Advanced Education in General Dentistry Program, Yakima, WA USA; 6 School of Dentistry, School of PublicHealth, University of Washington, Seattle, USA

**Keywords:** Mixed-dentition, Prediction equation, Tanaka and Johnston, Kenyan population

## Abstract

**Background:**

The Tanaka and Johnson equation is commonly used in mixed dentition analysis. However, the analysis is based on  a Caucasian population making clinical decisions challenging when used in different ethnic groups. This study developed a prediction equation based on a Kenyan population.

**Design:**

A descriptive cross-sectional study done in 68 13–17 years old Kenyans of African descent in two boarding secondary schools. Alginate impressions were taken, study models obtained, and mesiodistal tooth-widths measured on upper and lower study models from the first molar to the contralateral first molar. Descriptive statistics, paired t-tests and independent t-tests were conducted and Pearson product-moment correlation coefficients calculated (p < 0.05).

**Results:**

The mean age was 13.78 years (SD ± 0.70), females were 59%. The mesiodistal tooth-widths of the permanent canines and premolars were different between males and females (p ˂ 0.1). The Tanaka and Johnston equation significantly under-estimated the mesiodistal tooth-widths of the permanent canines and premolars (p ˂ 0.05). The addition of lower first permanent molars to the permanent lower incisors provided higher correlation coefficients than the Tanaka Johnston equation.

**Conclusions:**

A new equation that includes the permanent lower incisors and first permanent molars as predictor teeth seems to be more suitable for mixed dentition analysis for this Kenyan population. A larger study is needed to validate these findings.

## Background

Mixed dentition analysis is carried out to estimate the mesiodistal tooth-widths of unerupted permanent teeth for the purpose of making orthodontic treatment plans for the growing child [1, 2]. The methods used to estimate the space of the unerupted permanent canines and premolars include, among others, radiographic analysis, regression/prediction equations (using dental casts) or a combination of the two methods [3]. Although radiographic assessments are among the most accurate methods, such analyses require standardization and specialized equipment, which may not be widely available [4]. Prediction equations, mathematical formulae used to estimate the mesiodistal tooth-widths of unerupted canines and premolars, are commonly used because no special equipment is needed and can be easily applied [3]. These analyses usually employ erupted lower permanent incisors as the predictor teeth [5], with Tanaka and Johnston the most frequently utilized [1, 6].

The Tanaka and Johnston analysis is based on Caucasian populations [1, 6–8]. In this analysis, the combined mesiodistal tooth-widths of maxillary permanent canines and premolars in one quadrant is calculated by dividing the sum of the lower permanent incisors by two and adding eleven millimetres. In the case of the lower dental arch, the combined mesiodistal tooth-widths of mandibular permanent canines and premolars in one quadrant is calculated by dividing the sum of the lower permanent incisors by two and adding ten and a half millimetres. While it continues to be used worldwide, racial and ethnic differences have shown challenges when the Tanaka and Johnston analysis is applied to other populations besides Caucasians [9, 10].

The aim of this study was to develop an analysis tool that better predicts space for the Kenyan population. We hypothesized that there is a significant difference in the actual and calculated values of the sum of mesiodistal tooth-widths of the permanent canines and premolars using the Tanaka and Johnston equation compared to our analysis. We also hypothesized that the use of the lower permanent first molars in the estimate would give more accurate predicted mesiodistal tooth-widths of the permanent canines and premolars [11]. Results from this pilot study will be used in a larger project aimed at validating our proposed space analysis equation to be used by Kenyan oral health providers.

## Methods

This was a descriptive cross-sectional study carried out on Kenyan adolescents of African descent (aged 13–17 years old), attending two boarding secondary schools: Starehe Boys’ Centre and Starehe Girls’ Centre. Accounting for up to 10% of missing data and with a power of 80%, a study population of 68 subjects provided us the ability to detect effect sizes of 0.01 mm. Starehe Boys’ Centre is located about 3 km from Nairobi’s central business district in a low socio-economic status neighbourhood. Starehe Girls’ Centre is located about 15 km from Nairobi’s central business district in a peri-urban area. Students from the entire country are enrolled in these two schools. The two schools were chosen because of their large student body in Nairobi using convenient sampling method. The age range of 13–17 years was chosen to allow for only children in permanent dentition to be included. Initially, the study participants were selected through the school records that were used to identify the age, and thereafter a table of random numbers was then utilized to randomly select the participants to obtain the required sample size. Initial examinations were conducted among 400 boys and 200 girls, as a result of the greater student population in the Starehe Boys’ Centre. Only the study participants who fulfilled the inclusion criteria, which were (1) Kenyans of African descent, (2) fully erupted permanent incisors, canines, premolars and molars without morphological anomalies, (3) free of dental caries, (4) without clinical interproximal restorations, (5) without crown fractures, and (6) otherwise healthy, were included in the study.

### Data collection

The principal investigator (PI) was calibrated by an experienced orthodontist from the University Of Nairobi School Of Dental Sciences. Calibration was conducted using five pairs of study models of teeth in permanent dentition obtained from the Department of Pediatric Dentistry, University of Nairobi School of Dental Sciences. The data collection instrument, digital vernier caliper (Masel, USA), was first calibrated by the Kenya Bureau of Standards before use in this study. The intra-class coefficient to check for intra-examiner variation was 0.9 and for inter-examiner variation during calibration was 0.9 (Kappa score). Five pairs of randomly selected study casts were used to calculate the intra-class coefficient.

Dental impressions for data collection were completed in the health clinic of the girls’ school under natural light with the study participants seated on a normal chair. Similarly, same procedures were repeated for the boys’ school but on a dental chair in the dental clinic (available at the school premises). Irreversible hydrocolloid (alginate, Blueprint-Dentsply DeTrey GmbH [12], Germany) impressions (the material available to the study at the time) were obtained for both maxillary and mandibular arches using metallic dentate impression trays. Following the manufacturer’s recommendations, dental impressions made were rinsed under tap water, wrapped with moist gauze, and sealed in a polythene bag until they were poured in the dental laboratory of the School of Dental Sciences, University of Nairobi [12]. Transportation of the impressions took approximately one hour (due to the distance between the schools and laboratory), and samples were cast within 15 min of being received. This time frame was consistent with the manufacturer’s instructions to pour the moist and sealed alginate impressions [12]. A trained dental technician cast the impressions using type III dental stone using the recommended powder to water ratio, while vibrating the impression tray to eliminate air bubbles [12]. Each participant’s pair of study casts was then given a serial number corresponding to the serial number on the data capture form.

Following guidelines by Ngesa [13], who measured mesiodistal tooth-widths in a Kenyan population, each tooth was measured twice under natural light to the nearest 0.01 mm according to the digital vernier caliper utilized and the average value was then recorded. To minimize fatigue, only 3 pairs of dental casts were measured each hour. The PI conducted all measurements, including measuring twice five randomly selected casts for each gender one week apart to check for intra-examiner variability. Mesiodistal tooth-widths for both maxillary and mandibular arches were measured from the distal contact point of the first permanent molar to the distal contact point of the contralateral first permanent molar. A digital vernier caliper (MASEL, USA) was used to measure the mesiodistal tooth-widths. The beaks of the digital vernier caliper were placed perpendicular to the occlusal plane at the contact points of each tooth as described by Hunter and Priest [14]. Each tooth was measured twice to the nearest 0.01 mm and the average measurement was then entered in the data capture form which also contained the age and sex of the participant.

### Data analysis

Data capture paper forms were utilized to collect data for each study participant, and then transferred to a Microsoft Excel file. Each study subject received a study ID number. De-identified data was then coded and transferred to Statistical Package for Social Sciences (SPSS) software for analysis. Demographic data was analyzed with descriptive statistics. Paired t-test assessed statistically significant differences between the measured sum of mesiodistal tooth-widths of the permanent canines and premolars with the values expected when using the Tanaka and Johnston equation. Correlation coefficients were calculated to evaluate associations between the lower permanent incisors (predictor teeth) and the combined sum of mesiodistal tooth-widths of the permanent canines and premolars in one quadrant. Correlation coefficients were also calculated for the lower permanent incisors and first mandibular molars (predictor teeth) and the combined sum of mesiodistal tooth-widths of the mandibular permanent canines and premolars for both quadrants as described by Melgaco et al. [11]. Based on the correlation coefficients obtained, new prediction equations were formulated using the higher correlation coefficients. Higher correlation coefficients above 0.6 demonstrate a better association between the predictor teeth and the teeth whose mesiodistal tooth-widths are being predicted [3]. The level of significance was p ≤ 0.05.

## Results

Sixty-eight adolescents [13–17 years old] participated in the study. The majority of our study population was female (59%) with a mean age of 13.78 years ( ± 0.70) and a median age of 14 (Interquartile ratio (IQR): 1) (Table 1). Size of teeth varied by sex with boys having statistically significant larger mandibular and maxillary canines and premolars than girls (p < 0.01). We did not find any significant difference for the mandibular incisors (p = 0.27) (Table 2).

**Table 1:** Demographics of the study population

Sex	N (%)
Males	28 (41%)
Females	40 (59%)
Age: Mean (SD)	13.78 (± 0.70)
Age: Median (IQR)	14 (1)

^*^ SD: standard deviation, IQR: interquartile

**Table 2:** Mean mesiodistal tooth-widths in millimeters (mm) by sex of the study population

Teeth	Females Mean (mm ± SD)	Males Mean (mm ± SD)	p-value
Mandibular canines and premolars	20.90 ± 1.26	21.94 ± 1.63	< 0.01*
Maxillary canines and premolars	21.18 ± 1.16	22.25 ± 1.16	< 0.01*
Mandibular incisors	21.24 ± 1.35	21.66 ± 1.63	0.27

Independent T-test

*p ≤ 0.05

Measurements of our female study maxillary (p < 0.001) and mandibular (p = 0.19) canines and premolars were consistently smaller than values predicted by Tanaka Johnston. Among males, there were statistically significant differences between the measured values of permanent canines and premolars in our study population and those predicted by the Tanaka and Johnston equation mandible and maxilla (p = 0.05; 0.03; respectively) (Table 3).

**Table 3:** Comparison of measured sum of mesiodistal tooth-widths in millimetres (mm) with predicted values

	Females mean (mm ± SD)		Males mean (mm ± SD)	
	Mandibular canines and premolars	Maxillary canines and premolars	Mandibular canines and premolars	Maxillary canines and premolars
Actual (measured) ± SD	20.90 ± 1.26	21.18 ± 1.16	21.94 ± 1.63	22.25 ± 1.16
Tanaka and Johnston ± SD	21.12 ± 0.82	21.62 ± 0.8	21.33 ± 0.68	21.83 ± 0.68
*p value	0.19	0.00	0.05	0.03

Paired T-test

*SD* standard deviation

*p ≤ 0.05 is significant

Higher correlation coefficients (r) were obtained when the first permanent mandibular molars were added to the mandibular incisors and used as predictor teeth. The r values for the mandibular arch for females and males were 0.674 (compared to 0.550) and 0.652 (compared to 0.339), respectively. Similarly, the r values of the maxillary arch for females and males were 0.718 (compared to 0.630) and 0.725 (compared to 0.578), respectively (Table 4).

**Table 4:** Correlation coefficients of different groups of predictor teeth

Predictor teeth	Correlation coefficient (r) values			
	Females		Males	
	Mandibular arch	Maxillary arch	Mandibular arch	Maxillary arch
Four mandibular incisors	0.550	0.630	0.339	0.578
Four mandibular incisors and two first mandibular permanent molars	0.674	0.718	0.652	0.725

## Discussion

The Tanaka and Johnston equation is a widely used prediction equation although based on a Caucasian population [1, 13]. Due to the variability in tooth size due to ethnic and racial differences it has been shown to under/overestimate the mesiodistal tooth-widths of permanent canines and premolars [4, 5]. In this study, the Tanaka and Johnston equation was shown to under-estimate the mesiodistal tooth-widths of the permanent canines for both males and females. Buwembo et al. in a Ugandan study of 220 school children aged 12–17 years measured mesiodistal tooth-widths of incisors, canines and the premolars and showed that the Tanaka and Johnston equation overestimated tooth size for that population [13]. Alzubir et al. in a Sudanese study of 250 school children aged 13–19 years measured the mesiodistal tooth-widths of incisors, canines, premolars and the first molars and also showed that the Tanaka and Johnston equation overestimated tooth size for that population [4]. Giri et al. in a Nepalese study and several Indian studies (Ravinthar and Gurunathan, Bhatnagar et al. and Kakkar et al.) that measured mesiodistal tooth-widths on study casts also demonstrated the inaccuracy of the Tanaka and Johnston Eq. (7, 14–16).

For a prediction equation to be clinically useful according to Luu et al., the correlation coefficient (r) should be above 0.6 [3]. In this study, when only the four mandibular incisors were used as predictor teeth, correlation coefficients below 0.6 were obtained. This means that the estimated mesiodistal tooth-widths of the permanent canines and premolars were not close to the actual tooth-widths. Our results showed that mixed dentition analysis done using these estimates will be inaccurate and lead to erroneous treatment plans for growth and development. To overcome this deficiency, the method developed by Melgaco et al. [9] was used to obtain clinically useful correlation coefficients above 0.6. They included the first mandibular permanent molar as a predictor tooth and obtained correlation coefficients of 0.795–0.81, which were similar to those obtained by radiographic methods (0.84) by Hixon and Oldfather [17]. Using this method, correlation coefficients obtained were 0.674 (females) and 0.718 (males) for the mandibular arch and 0.653 (females) and 0.725 (males) for the maxillary arch. Thus, values obtained using this method were closer to the actual values, compared to using the Tanaka and Johnston method and can be used effectively during mixed dentition analysis with minimal errors. Mittal et al. used the Melgaco et al. method on conventional study models and obtained a correlation coefficient of 0.957 [18]. Shahid et al. used digital study models and obtained correlation coefficients of 0.7395–0.85994 [19]. Study findings of this study therefore corroborate well with these other studies using the Melgaco et al. method.

This study had several limitations. Firstly, it was the low sample size (68) compared to other similar studies [2, 4, 13]. Nonetheless, this is the very first pilot study on the topic among a  Kenyan population. Secondly, the time that elapsed between taking the alginate impressions and making of the casts. While not immediately, the impressions were not poured within 15 min due to the time lag of transporting the impressions to the appropriate laboratory for processing. However, we followed the manufacturer's protocol for pouring the impressions. [10]. Additionally, there are similar studies (Buwembo et al, Alzubir et al and Giri et al.) that have also used study models poured after the recommended manufacturer time and they also demonstrated the inaccuracy of the Tanaka and Johnston equation  (4, 7, 13). Ideally, we would have hoped to use a digital impression taking technique but this was not possible at the time of the study due to lack of access to such technology and funding.

This was a pilot study to investigate the accuracy of the Tanaka and Johnston equation in a Kenyan population and develop a more accurate prediction equation. Further studies should validate current results using larger sample sizes to provide more accurate prediction  equations for Kenyan population in the mixed dentition stage.

## Conclusions

The Tanaka and Johnston equation was not accurate in estimating the mesiodistal tooth-widths of the permanent canines and premolars for this Kenyan study population. However, the inclusion of the first mandibular permanent molar as a predictor tooth resulted in high correlation coefficients.

## Data Availability

The datasets used and/or analysed during the current study are available from the corresponding author on reasonable request.
